# Advanced and Metastatic Gastrointestinal Stromal Tumors Presenting With Surgical Emergencies Managed With Surgical Resection: A Case Series

**DOI:** 10.7759/cureus.53851

**Published:** 2024-02-08

**Authors:** Divij Jayant, Mrinal Goyal, Vipul Thakur, Swapnesh Sahu, Basil Babu, Satish Subbiah Nagaraj, Cherring Tandup, Arunanshu Behera

**Affiliations:** 1 General Surgery, Postgraduate Institute of Medical Education and Research, Chandigarh, IND

**Keywords:** damage control laparotomy, imatinib mesylate, bowel obstruction, bleeding gist, surgical emergency scenarios, gastrointestinal stromal tumor (gist)

## Abstract

Advanced and metastatic gastrointestinal stromal tumors (GISTs) presenting with surgical emergencies are rare. Neoadjuvant imatinib being the treatment of choice for non-metastatic advanced disease with a proven role in downstaging the disease may not be feasible in patients presenting with bleeding and obstruction.

We present a case series with retrospective analysis of a prospectively maintained database of patients with advanced and metastatic GISTs presenting with surgical emergencies. Clinical characteristics, imaging and endoscopic findings, surgical procedures, histological findings, and outcomes in these patients were studied. Four patients were included in this case series, with three males and one female (age range: 24-60 years). Two patients presented with melena; one with hemodynamic instability despite multiple blood transfusions underwent urgent exploratory laparotomy for bleeding gastric GIST, while the other underwent surgical exploration after careful evaluation given the recurrent, metastatic disease with a stable metabolic response on six months of imatinib. One patient with metastatic jejunal GIST who presented with an umbilical nodule and intestinal obstruction was given a trial of non-operative management for 72 hours, but due to non-resolution of obstruction, segmental jejunal en bloc resection with the dome of the urinary bladder with reconstruction and metastasectomy was needed. The patient with advanced gastric GIST who presented with gastric outlet obstruction was resuscitated, and an attempt of endoscopic naso-jejunal tube placement was tried, which failed, and exploration was needed. The mean length of hospital stay was 7.5 days. Histopathological examination confirmed GIST in all four patients with microscopic negative resection margins. All patients were started on imatinib with dose escalation to 800 mg in the patient with recurrent and metastatic disease; however, the patient with bleeding gastric GIST experienced severe adverse effects of imatinib and discontinued the drug shortly. All four patients are disease-free on follow-ups of 15 months, 48 months for the patient with advanced non-metastatic disease, and six and 24 months for the patients with metastatic disease. In the era of tyrosine kinase inhibitor (TKI) therapy for advanced and metastatic disease, upfront surgery is usually reserved for surgical emergencies only. Surgical resection, the cornerstone for the treatment of resectable GIST, may also be clinically relevant in metastatic settings, although it requires a careful and individualized approach.

## Introduction

Gastrointestinal stromal tumors (GISTs) can occur throughout the gastrointestinal tract and account for up to 1%-3% of malignant tumors affecting the gastrointestinal tract [1]. Most frequently occurring within the stomach and small intestine, 20%-30% have metastatic disease at presentation [2]. Presenting with symptoms based on the tumor site [3], patients usually complain of vague abdominal discomfort (60%-70%), followed by bleeding (30%-40%). Bleeding, obstruction, and tumor perforation are rare surgical emergencies. Diagnosis requires immunohistochemistry of the tumor and genetic mutation analysis. The majority are positive for c-KIT (CD117) (95%) and DOG1 (98%) [4]. Up to 75% of GISTs have KIT mutations with exon 9 (8%), exon 11 (90%), exon 13 (1%), and exon 17 (1%), while 10%-20% have platelet-derived growth factor alpha gene (*PDGFRA*) mutation [5]. Before the advent of tyrosine kinase inhibitors (TKIs), up to 50% of patients experienced recurrence or metastasis even after complete resection [6]. The use of imatinib mesylate has improved the prognosis of metastatic and recurrent disease [7]; however, upfront surgery for locally advanced disease can lead to extensive R0 resection and add to overall morbidity, which may be unnecessary as the use of neoadjuvant TKIs in this setting has been reported to allow less extensive surgery, thereby preserving surrounding organs as a result of tumor shrinkage [8,9]. Although upfront surgery for metastatic disease is reserved for emergencies such as bleeding, obstruction, and compressive symptoms, cytoreductive surgery or even R0 resection, if possible, can be life-saving with the added benefit of post-debulking imatinib effect. Alternatively, locally advanced and metastatic disease presenting as a surgical emergency may not be feasible for pre-operative imatinib therapy. Thereby, such cases require careful pre-operative planning regarding resectability and balance between oncological resection and surgery for symptomatic palliation in an otherwise metastatic disease that might not be resectable.

We present a case series of four patients with locally advanced, recurrent, and metastatic GIST of the stomach and small bowel presenting with surgical emergencies and managed with surgery and received adjuvant TKIs. We also discuss the oncological goals and management principles in emergency conditions.

## Case presentation

Methods

This is a case series with a retrospective analysis of a prospectively maintained database on non-consecutive patients presenting to the surgical department of Postgraduate Institute of Medical Education and Research, Chandigarh, India, with advanced, metastatic, and recurrent GIST presenting with surgical emergencies managed with surgical resection from July 2018 to June 2023. A total of four patients met the abovementioned criteria and were included in the case series. We describe the clinical presentation, imaging diagnostic features, intra-operative images, surgical procedures, and outcomes of these patients.

The data collected included patient demographics, symptomatology, abdominal signs, anatomical localization, tumor characteristics, laboratory findings, surgical procedure performed, pathological findings, immunohistochemical (IHC) analysis, length of hospital stay, and intra-operative and post-operative complications. Pre-operative diagnosis and evaluation of tumor characteristics were performed using upper GI (UGI) endoscopy and contrast-enhanced computed tomography (CECT). Patients were operated on via open technique. Intra-operatively, the tumor was resected with adequate margins (>1 cm) and without tumor capsule breach. Pathological specimens were examined for tumor site, size, margins, and serosal and mucosal breach and histologically analyzed for tumor subtype, nature, mitotic rate, tumor necrosis, lymphovascular invasion, and tumor grade. IHC analysis was performed using markers such as Ki67%, CD117, c-KIT, DOG1, and S-100 protein. Data was reported as nominal, ordinal, discrete, and continuous variables. All patients were followed up with length of hospital stay, routine visits every two weeks, days after discharge till three months, monthly follow-up till six months, third monthly follow-up till two years, and six monthly follow-up thereafter with clinical examination and CECT of the abdomen.

A literature review was performed for previous publications on PubMed and Google Scholar databases, using the terms "locally advanced, recurrent, and metastatic GIST," "GI bleed," "endoscopy," and "surgery." The study has been reported in line with the Preferred Reporting Of CasE Series in Surgery (PROCESS) guidelines [10].

Results

The case details are summarized in Table 1. A total of four patients, three males and one female, with ages ranging from 24 to 60 years, were studied. All the patients had a history of abdominal pain and a lump on the abdomen on examination.

**Table 1:** Patient demographics, clinical presentation, radiology, intra-operative findings, and outcomes. Abbreviations: UGI: upper gastrointestinal tract, CECT: contrast-enhanced computed tomography, FDG-PET: fluorodeoxyglucose positron emission tomography, FNAC: fine-needle aspiration cytology, LOHS: length of hospital stay, POPF: post-operative pancreatic fistula, TKI: tyrosine kinase inhibitor

Case details	Patient1	Patient 2	Patient 3	Patient 4
Age	60 years	49 years	24 years	33 years
Sex	Male	Male	Male	Female
Abdominal pain	No	Yes	Yes	Yes
Melena/hematemesis	No	No	Yes	Yes
Intestinal obstruction	Yes	No	No	No
Lump per abdomen	Yes	Yes	Yes	Yes
Early satiety/gastric outlet obstruction	No	Yes	Yes	Yes
Previous history	No	No	9 years ago, segmental jejunal resection	3 months ago, melena
Blood transfusion and number	No	No	3 units	6 units
UGI endoscopy	Normal	Extrinsic compression from the body to the antrum and pylorus of the stomach	Polypoidal lesion at the body of the stomach with active bleeding	Large pedunculated polypoidal lesion on the fundus and body with active bleeding
Multiphasic CECT of the abdomen	10 × 9 × 9 cm ileal mass, loss of fat planes with urinary bladder, with proximal small bowel dilatation	21 × 12 × 10.5 cm gastric mass, abutting the left hemidiaphragm and the body and tail of the pancreas	13 × 92 × 90 cm left subdiaphragmatic, posteroinferior to the spleen and indenting the stomach with loss of fat planes at the body, omental 25 × 21 × 16 mm nodule with central necrosis	13 × 12 × 8 cm mass in the fundus and body of the stomach with hyperdense contents in the stomach lumen
FDG-PET	10 × 12 × 11 cm ileal mass, loss of fat planes with urinary bladder, multiple nodules at the umbilicus, mesentery, and omentum	No	No	No
Preoperative FNAC/biopsy	Yes, umbilical nodule suggestive of spindle cell tumor	Inconclusive	Yes	No, hemodynamically unstable
Surgery	Open exploratory laparotomy	Open exploratory laparotomy	Open exploratory laparotomy	Open exploratory laparotomy
Indication	Intestinal obstruction	Gastric outlet obstruction	Bleeding	Bleeding
Procedure	Jejunal segmental en bloc resection with the dome of the urinary bladder, omentectomy, excision of multiple mesenteric deposits	En bloc resection of the left hemidiaphragm, sleeve resection of the body of the stomach with distal pancreaticosplenectomy	En bloc resection of the left hemidiaphragm with sleeve gastrectomy, splenectomy, and omentectomy	Subtotal gastrectomy with feeding jejunostomy Roux-en-Y esophago-jejunal pouch anastomosis
Intra-operative findings	15 × 12 cm jejunal mass, 100 cm distal to the duodenojejunal flexure infiltrating the dome of the urinary bladder, omphalectomy, excision of multiple omental and mesenteric deposits	25 × 15 × 15 cm gastric mass from the posterior surface of the body of the stomach infiltrating the left hemidiaphragm, pancreatic body, and tail	15 × 10 cm retroperitoneal mass infiltrating the left hemidiaphragm and the posterior wall of the stomach and spleen, abutting the pancreas with single omental deposit	12 × 12 cm intraluminal mass with exophytic posterior extension present with active bleeding
Post-operative complications	Prolonged per urethral Foley catheter requirement for 7 days	POPF grade A: biochemical leak	None	Gastric stump blowout managed with ultrasound-guided pigtail drainage of collection
LOHS	8 days	5 days	5 days	12 days
Adjuvant TKI use	Imatinib 400 mg OD	Imatinib 400 mg od	Imatinib 400 mg bd	Not taken
Follow-up	24 months, asymptomatic, no recurrence till date	15 months, asymptomatic, no recurrence till date	6 months, asymptomatic, no recurrence till date	48 months, asymptomatic, no recurrence till date

Patient 1 presented with intestinal obstruction, which was managed with Ryles tube decompression and intravenous fluid resuscitation. He had a recurrent history of abdominal fullness and colicky abdominal pain after meals, which was being managed with oral acetaminophen and oral hyoscine prescribed by the local practitioner. A trial of non-operative management for 72 hours was given to the patient, but there was no symptomatic improvement. Patient 2 presented with early satiety and vomiting after intake of meals, which had been gradually increasing for six months with significant loss of appetite and weight. Patients 3 and 4 presented with hematemesis and melena to surgical emergency with hypotension.

Patient 3 had presented with melena nine years ago and on evaluation was diagnosed with a bleeding jejunal GIST, which was managed with surgical resection of GIST at duodenojejunal flexure and anastomosis. With biopsy-proven high-grade GIST, the patient was advised with adjuvant imatinib, which was taken for three years, following which the patient was lost to follow-up. The patient presented with abdominal pain and early satiety with significant loss of appetite and weight for six months after six years. An upper gastrointestinal (UGI) endoscopy was suggestive of extrinsic compression at the body and antrum, and fine-needle aspiration cytology (FNAC) was suggestive of spindle cell tumor. Genetic analysis was not available at our institute. Given the recurrence of the disease with single omental metastasis on CECT, imatinib was restarted and continued for six months when the patient presented to surgical emergency again with vomiting and melena. Hemoglobin was 5 gm/dL on presentation, and a UGI endoscopy was evident of active bleeding from the lesion on the body of the stomach. Contrast-enhanced computed tomography (CECT) revealed that the size of the primary lesion had increased (11 × 9.2 × 9 cm from 9.7 × 7 × 7.4 cm), but the size of omental metastasis (2.5 × 2.1 × 1.6 cm from 5 × 4.4 × 3.5 cm) had decreased. The patient was planned for exploration because of active bleeding.

Patient 4 had a previous history of melena three months prior when she was advised an upper gastrointestinal (UGI) endoscopy, but the patient refused. On presentation, hemoglobin was 2.9 gm/dL, and the UGI endoscopy revealed a large polypoidal lesion in the fundus and body with active bleeding. The patient required urgent laparotomy because of active bleeding and hemodynamic instability.

**Figure 1:**
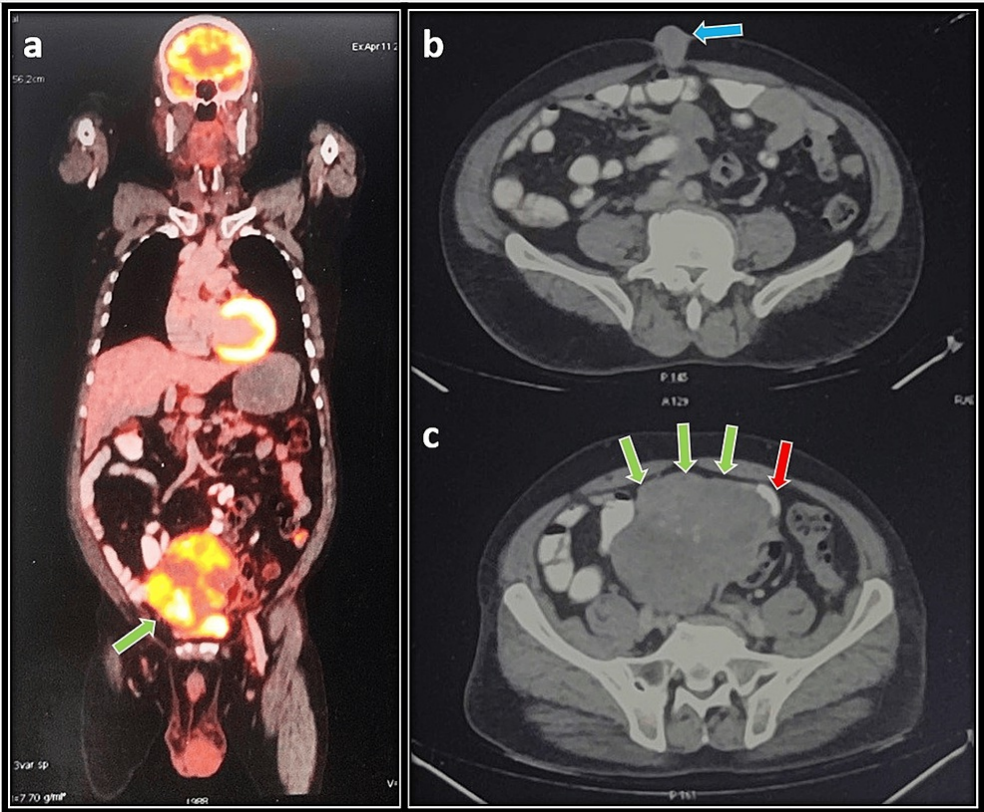
(Patient 1) a (green arrow): FDG-PET reconstructed image showing an enhancing mass lesion arising from the small bowel infiltrating the dome of the urinary bladder. b (blue arrow): Metastatic enhancing lesion at the umbilicus presenting as an umbilical nodule. c (green arrows): GIST arising from small bowel loops (red arrow). FDG-PET: fluorodeoxyglucose positron emission tomography, GIST: gastrointestinal stromal tumor

**Figure 2:**
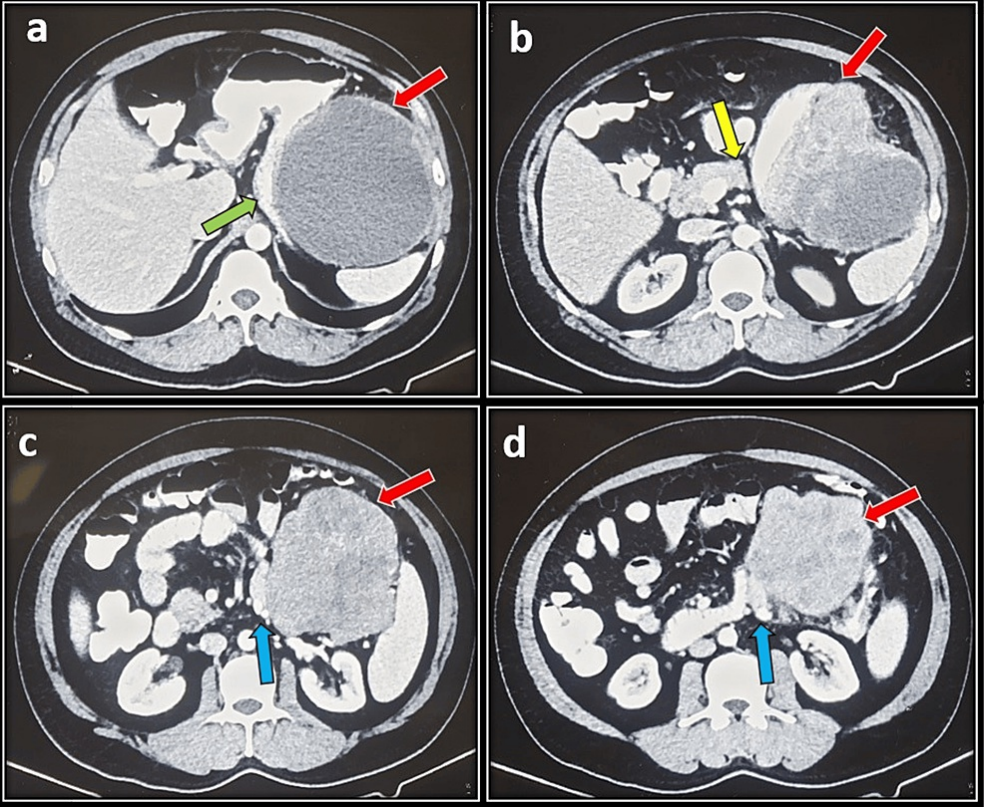
(Patient 2) a (red arrow): Solid cystic heterogenous enhancing lesion compressing the proximal stomach (green arrow) causing gastric outlet obstruction. b (red arrow): Tumor infiltrating inferiorly the body and tail of the pancreas (yellow arrow). c (red arrow): Heterogenously enhancing tumor causing a mass effect in the duodenojejunal flexure with resultant medial displacement (blue arrow). d (red arrow): Heterogenously enhancing tumor with suspicious infiltration of the proximal jejunum (blue arrow).

On endoscopic evaluation, only patient 1 had a normal UGI endoscopy, while patient 2 had extrinsic compression from the body to the antrum and pylorus, and patients 3 and 4 had active bleeding from the polypoidal lesion at the body and active bleeding from the pedunculated lesion at the fundus and body, respectively. CECT of the abdomen revealed a jejunal mass in patient 1 (Figure 1), gastric mass in patients 2 (Figure 2) and 4, and left subdiaphragmatic mass inferior to the spleen infiltrating the body of the stomach in patient 3. The tumor size range was from 10 to 21 cm. Fluorodeoxyglucose positron emission tomography (FDG-PET) was only available in patient 1, which was evident of small bowel mass with urinary bladder infiltration and multiple mesenteric, omental deposits and umbilical nodule (Figure 1). Preoperative FNAC was available in patients 1 and 3, which was suggestive of spindle cell tumor with positive c-KIT, while FNAC was inconclusive in patient 2 and was not done in patient 4 due to hemodynamic instability. The indication of surgery was intestinal obstruction in patient 1, gastric outlet obstruction in patient 2, and active bleeding in patients 3 and patient 4.

**Figure 3:**
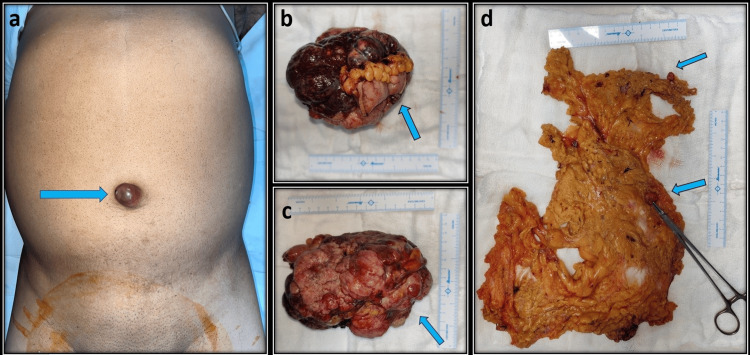
(Patient 1) a (blue arrow): Metastatic jejunal GIST presenting with an umbilical nodule. b (blue arrow): Approximately 15 × 15 cm tumor arising from the jejunum with urinary bladder infiltration. c (blue arrow): Lobulated tumor with areas of hemorrhage and necrosis. d: Omentectomy specimen showing multiple omental tumor deposits (blue arrows). GIST: gastrointestinal stromal tumor

All patients underwent open exploratory laparotomy due to the advanced nature of the disease presenting as a surgical emergency. Patient 1 required en bloc resection of jejunal GIST with the dome of the urinary bladder along with resection of omental and mesenteric deposits and excision of the umbilical nodule with adequate margins (>1 cm) (Figure 3). Patient 2 required en bloc resection of the left hemidiaphragm, sleeve resection of the body of the stomach, and distal pancreaticosplenectomy. Patient 3 required en bloc resection of the left hemidiaphragm with sleeve gastrectomy, splenectomy, and omentectomy, while patient 4 underwent a staged procedure with subtotal gastrectomy and feeding jejunostomy during the first surgery. Reconstruction was deferred at that time because of hemodynamic instability. Four weeks later, Roux-en-Y esophago-jejunal pouch anastomosis was done. Patients 1, 2, and 3 had uneventful post-operative courses with a mean length of hospital stay of 7.5 days (range: 5-12 days). Patient 4 developed gastric stump blowout, which was managed with ultrasound-guided pigtail drainage of collection, and was discharged on the 12th post-operative day. Histopathological and IHC analyses were performed according to Fletcher criteria and confirmed GIST in all four cases (Table 2). Tumor sizes ranged from 11.5 to 20 cm in maximum dimension and were in accordance with radiological findings (Table 2). Patients 1, 2, and 4 had high-grade GIST, while patient 3 had moderate-risk GIST. All the lesions had microscopically negative resection margins with mucosal breech in patients 1, 2, and 4 and serosal breech in patient 1. Patients 1 and 2 were started on adjuvant oral imatinib 400 mg daily. On follow-up of 24 months, patient 1 is disease-free, and patient 2 is 15 months disease-free at follow-up. Patient 3 was again started on oral imatinib at 800 mg daily and is 12 months disease-free at follow-up. Patient 4 was also advised imatinib but developed diarrhea and generalized edema and discontinued the drug after a week; presently, patient 4 is 48 months disease-free on follow-up without adjuvant TKI.

**Table 2:** Tumor characteristics on histopathological examination. GIST: gastrointestinal stromal tumor, hpf: high-power field

	Patient 1	Patient 2	Patient 3	Patient 4
Tumor type	Jejunal GIST	Gastric GIST	Jejunal GIST	Gastric GIST
Tumor size	15 × 10.2 × 8 cm	20 × 15 × 12 cm	12 × 8.5 × 7 cm	11.5 × 9 × 5.5 cm
Nature	Spindle cell	Spindle cell	Spindle cell	Spindle cell
Mitotic rate	27/50 hpf	25/50 hpf	<5/50 hpf	>15/50 hpf
Tumor grade	High risk	High risk	Moderate	High risk
Margins	Free	Free	Free	Free
Mucosal breach	Yes	Yes	No	Yes
Serosal breach	Yes	No	No	No
CD117/c-KIT	Positive	Positive	Positive	Positive
DOG1	Positive	Positive	Positive	Positive
S-100	Negative	Negative	Negative	Negative
Ki67%	>20%	>20%	>10%	>10%
Metastasis	Yes, multiple omental, mesenteric deposits	No	Yes, single omental deposit	No

## Discussion

GISTs are the most common mesenchymal tumors affecting the gastrointestinal tract [11]. Mostly affecting the elderly with a slight male predisposition, at a mean age of 60-65, the younger population affected may be part of the non-hereditary Carney triad or autosomal dominant Carney-Stratakis syndrome with predisposing germline SDH mutations [12,13]. GIST occurring in children and adults <30 years old arises mostly in the stomach [14]. In our case series, the youngest patient, at 15 years of age, was diagnosed with and managed for a bleeding jejunal GIST and presented with a recurrence nine years later. However, no associating syndrome in any of the patients in our case series was detected. GIST usually presents as a well-defined submucosal or subserosal mass in the stomach (60%), followed by the small intestine (25%), and less often in the colon, rectum, esophagus, mesentery, and omentum [15]. The clinical presentation varies according to tumor location and size, and although the majority are asymptomatic and discovered incidentally [15] on imaging, symptomatic GISTs most commonly present with abdominal pain and hematemesis or melena due to bleeding [16]. Up to 10% of gastric and 15% of duodenal GISTs can present with UGI bleeding. Small intestinal (jejunal: 40%, ileal: 60%) GISTs may present with small bowel bleeding, fatigue (due to anemia), abdominal pain, and intestinal obstruction [17]. At diagnosis, 20%-30% of GIST are metastatic, most commonly in the abdomen, liver, lungs, and bones. Lymph node metastasis is rare, except in pediatric-type GIST in young adults and GIST associated with clinical syndromes [18]. In our case series, two patients with advanced gastric GIST presented with gastric outlet obstruction and bleeding, while in two patients, the one with metastatic jejunal GIST presented with an umbilical nodule and obstruction, and the other case of recurrent, metastatic jejunal GIST on imatinib therapy presented with UGI bleeding. Although pain in the abdomen and bleeding are the usual presentation in GIST, gastric outlet obstruction is rare, and a metastatic jejunal GIST presenting with an umbilical nodule is even rarer and has not been reported in the literature.

Not all GISTs show malignant behavior, but 20%-25% of gastric and 40%-50% of small intestinal GISTs do show clinically malignant behavior [19]. As was seen in our series, two patients with jejunal GIST were diagnosed to have metastatic disease at presentation, one of whom was started on imatinib therapy. Patients with large and locally advanced gastric GIST did not have any metastasis at the time of presentation.

Stomach and small bowel GIST on CECT may show an intramural endophytic or exophytic homogenous, smooth-walled, sharply marginated, hypervascular mass in the stomach or small bowel wall. Large (>5 cm) GIST may have a heterogenous appearance due to necrosis, hemorrhage, or cystic degeneration. In a patient presenting with acute GI bleed, multiphasic computed tomography angiography (CTA) is the recommended investigation of choice in hemodynamically stable patients, having an accuracy of 100% to localize bleeding at a rate of 0.3 mL/minute with a sensitivity of 85% [20]. Further, CT is also helpful in assessing tumors and their relationship with adjacent structures, follows the natural progression of GIST and response to TKI in cases of locally advanced and metastatic GIST, and at the same time has a faster acquisition time for surgical planning if needed [21].

On endoscopy, GIST appears as hemispherical, subepithelial lesions, and endoscopic ultrasound (EUS) is required to ascertain the layer of the GI wall from which they originate. Mostly originating from the muscularis propria (echo-poor fourth layer) and rarely from muscularis mucosae (echo-poor second layer), the differential diagnosis includes leiomyoma, lymphoma, and schwannoma, which requires tissue diagnosis with fine-needle aspiration (FNA) for >1 cm lesion and tru-cut biopsy for >2 cm lesion if neoadjuvant TKI therapy is planned for locally advanced and metastatic GIST [22]. On the other hand, in a symptomatic GIST with malignant features on EUS, such as size > 2 cm, irregular borders, heterogenous echogenicity, anechoic cystic space, ulceration, echogenic foci, marginal halo, and size increase on follow-up, tissue diagnosis is not mandatory, and patients may undergo upfront surgical resection [23]. Locally advanced and metastatic GIST should be investigated prior with a baseline FDG-PET. The stage of the disease, response to treatment, and resistance to treatment can be assessed objectively in addition to resolving any discrepancy from CT findings of inflammatory adhesions and contiguous organ involvement [24]. The patients in our case series presented with small bowel obstruction, bleeding, and gastric outlet obstruction and were resuscitated before any investigations were planned. Patients with UGI bleeding were resuscitated with blood products and required urgent endoscopy, which revealed a subepithelial polypoidal bleeding lesion. The patient who presented with small bowel obstruction had a normal UGI but had a dilated small bowel with evidence of multiple mesenteric and omental deposits on CECT, while the patient with gastric GIST who presented with gastric outlet obstruction only had an extrinsic compression on the body, antrum, and pylorus, and even FNA was inconclusive. Although a CECT was done before surgery in all patients in our series, the ability to anticipate and formulate a surgical plan for a metastatic jejunal GIST with small bowel obstruction, a large locally advanced gastric GIST with gastric outlet obstruction, a locally advanced and metastatic GIST with bleeding, and a large gastric GIST with bleeding before surgery was helpful for a favorable outcome.

Having an unpredictable biological behavior and resistance to traditional chemotherapy and radiotherapy, upfront surgery is curative in up to 60% of patients with localized and resectable GIST [25], but the risk of relapse and progression is also considerable [26]. Alternatively, surgery for metastatic GIST is safe, but its efficacy to prolong overall survival is unknown [27,28]. Before the discovery of TKI, the prognosis of patients with advanced disease was poor, with a one-year survival rate of 12% [29] and a median survival of 18-24 months [30], and although surgical resection of recurrent and metastatic GIST was associated with improved survival if R0 resection was achieved [31], the use of imatinib as adjuvant therapy for high-risk tumors improves overall survival [32]. Its use in the neoadjuvant setting is safe [33] and has been reported to have a role in downstaging disease; it allows R0 resection and allows local disease control in patients with locally advanced, marginally resectable disease in which upfront surgery may be technically challenging, morbid, or not feasible [8,9,34]. A 2009 review article by Fernández et al. proposed the use of surgery and imatinib therapy as a multimodal approach [35]. Similarly, in 2015, Chang et al. observed a higher rate of complete resections after initial imatinib therapy [36]. Meanwhile, in 2012, Tielen et al. retrospectively analyzed 57 patients and concluded that imatinib therapy increased the rates of R0 resections and reduced tumor perforations, subsequently improving patient outcomes [34]. Although the maximal response to imatinib is typically achieved within 6-18 months of treatment [37], despite its use, an improvement in R0 resection and overall survival has not been proven as of yet [38], nor is imatinib curative in the presence of residual, microscopic disease [6].

The rationale of surgery in metastatic GIST is based on the principle of reducing the number of pre-existing resistant clones, thus delaying tumor progression as imatinib has a complete response rate of less than 3% and a median progression-free survival of less than two years. Thus, in patients with limited disease, surgery may be curative as long as adjuvant TKI is continued after surgery [39]. Indications of cytoreductive surgery for recurrent and metastatic GIST are bleeding, intestinal perforation, obstruction, and patients with stable disease or TKI responders whenever the disease is resectable [40]. In a 2018 review of literature by Kikuchi et al., studying the various management options and optimal timing for surgery in metastatic GIST patients, they concluded that upfront cytoreductive surgery in metastatic cases has not shown any survival benefit and imatinib therapy remains the initial treatment of choice [41]. However, emergencies such as bleeding, obstruction, and compressive symptoms remain indications for upfront surgery even in cases of advanced disease [35,36,38,42,43]. In 2014, Sorour et al. observed that out of 92 GIST patients presenting to the emergency, 90 underwent emergency surgical resection for GIST-related complications. Out of the 90 surgical resections, 86 achieved complete/R0 surgical resection [42].

Similarly, in all the cases under consideration, surgery was deemed necessary due to presentation with surgical emergencies such as intestinal obstruction, UGI bleeding, and gastric outlet obstruction. In the patients with locally advanced disease who presented with UGI bleeding and gastric outlet obstruction, urgent surgical exploration was warranted for UGI bleeding due to hemodynamic instability; however, it needed a subtotal gastrectomy in the patient who was not hemodynamically stable for anastomosis at that time, and an abbreviated laparotomy with stapling of proximal stomach, insertion of Ryles tube just proximal to the stapled end, and a feeding jejunostomy was done. This was followed by Roux-en-Y jejunal pouch reconstruction and anastomosis after six weeks as following the first surgery on post-operative day 3, gastric stump blowout complicated the post-operative period, which was managed with the help of ultrasound-guided drainage. The other patient with a large locally advanced gastric GIST with gastric outlet obstruction in whom endoscopic nasoduodenal tube placement was not successful also needed upfront surgery with sleeve resection of the stomach, left hemidiaphragm, and distal pancreaticosplenectomy. Although extensive resection was needed in these patients, the plan for surgical exploration was considered only after the failure of endoscopic management. The patients with metastatic and recurrent GIST who presented with surgical emergencies created a dilemma regarding surgical planning. The metastatic jejunal GIST patient with intestinal obstruction was given a trial of non-operative management with nil per oral, Ryles tube decompression for 72 hours, but as there was no symptomatic improvement, persistent obstruction surgery was required. The other patient with recurrent, metastatic jejunal GIST on imatinib therapy for six months presented with UGI bleeding for whom surgery was also deemed necessary.

Complete (R0) resection was achieved in all the cases, and all the patients were started on TKI therapy soon after surgery. However, the patient with bleeding gastric GIST on adjuvant imatinib developed adverse effects with diarrhea and generalized edema and discontinued imatinib after a week. The patient was advised sunitinib but was not able to afford the same due to financial constraints. This patient is presently 48 months disease-free at follow-up without the use of any TKIs. Although the median time to recurrence after the resection of high-risk GIST is two years, survival benefit is seen in patients with a high risk of recurrence (mitotic count > 5/50 hpf, size >5 cm, non-gastric location, and tumor rupture) [27,29,35,39]. As per the current National Comprehensive Cancer Network (NCCN) guidelines, upfront surgery with or without adjuvant TKI is the treatment of choice for a surgically resectable or early disease. However, for locally advanced and metastatic burden of disease, the role of upfront surgery is reserved for emergent conditions such as uncontrolled tumoral bleed/perforation/rupture/obstructive or compressive symptoms causing significant pain or distress. Otherwise, any patient presenting with advanced/metastatic tumor should be initially treated with imatinib therapy (for imatinib-sensitive tumors) and planned for cytoreductive surgery once optimal response to the treatment has been achieved. The timing of such a cytoreductive procedure has been an issue of much debate and is usually about six months from the start of TKI therapy with case-to-case variation. Additionally, the benefit of such a cytoreductive procedure in a metastatic setting remains unclear [41]. However, there is a consensus that only macroscopically complete resection (R0/R1) has shown any survival benefit over the R2 resection/imatinib-only approach.

## Conclusions

In the era of TKI therapy, the role of surgery in the treatment of advanced GIST has been primarily adjuvant. However, acute abdomen and GI bleeding remain the main indications for upfront surgical resections in the case of advanced GIST. Even then, R0 resections are possible and, as such, lifesaving.
